# The role of spreading depolarizations and electrographic seizures in early injury progression of the rat photothrombosis stroke model

**DOI:** 10.1177/0271678X20915801

**Published:** 2020-04-02

**Authors:** Karl Schoknecht, Majed Kikhia, Coline L Lemale, Agustin Liotta, Svetlana Lublinsky, Susanne Mueller, Philipp Boehm-Sturm, Alon Friedman, Jens P Dreier

**Affiliations:** 1 Center for Stroke Research Berlin (CSB), Charité – Universitätsmedizin Berlin, corporate member of Freie Universität Berlin, Humboldt-Universität zu Berlin, and Berlin Institute of Health, Berlin, Germany; 2 Department of Neurology, Charité – Universitätsmedizin Berlin, corporate member of Freie Universität Berlin, Humboldt-Universität zu Berlin, and Berlin Institute of Health, Berlin, Germany; 3 Department of Experimental Neurology, Charité – Universitätsmedizin Berlin, corporate member of Freie Universität Berlin, Humboldt-Universität zu Berlin, and Berlin Institute of Health, Berlin, Germany; 4 Neuroscience Research Center, Charité – Universitätsmedizin Berlin, corporate member of Freie Universität Berlin, Humboldt-Universität zu Berlin, and Berlin Institute of Health, Berlin, Germany; 5 Institute for Neurophysiology, Charité – Universitätsmedizin Berlin, corporate member of Freie Universität Berlin, Humboldt-Universität zu Berlin, and Berlin Institute of Health, Berlin, Germany; 6 Carl-Ludwig-Institute for Physiology, Medical Faculty, University of Leipzig, Leipzig, Germany; 7 Einstein Center for Neurosciences Berlin, Charité – Universitätsmedizin Berlin, corporate member of Freie Universität Berlin, Humboldt-Universität zu Berlin, and Berlin Institute of Health, Berlin, Germany; 8 Department of Anesthesiology, Charité – Universitätsmedizin Berlin, corporate member of Freie Universität Berlin, Humboldt-Universität zu Berlin, and Berlin Institute of Health, Berlin, Germany; 9 Departments of Physiology & Cell Biology, Cognitive & Brain Sciences, the Zlotowski Center for Neuroscience, Ben-Gurion University of the Negev, Beer-Sheva, Israel; 10 NeuroCure Cluster of Excellence and Charité Core Facility 7T Experimental MRIs, Charité – Universitätsmedizin Berlin, corporate member of Freie Universität Berlin, Humboldt-Universität zu Berlin, and Berlin Institute of Health, Berlin, Germany; 11 Department of Medical Neuroscience, Dalhousie University, Halifax, Canada; 12 Bernstein Center for Computational Neuroscience Berlin, Humboldt-Universität zu Berlin, Germany

**Keywords:** Blood–brain barrier, ketamine, seizure, spreading depolarization, stroke

## Abstract

Spreading depolarization (SD) and seizures are pathophysiological events associated with cerebral ischemia. Here, we investigated their role for injury progression in the cerebral cortex. Cerebral ischemia was induced in anesthetized male Wistar rats using the photothrombosis (PT) stroke model. SD and spontaneous neuronal activity were recorded in the presence of either urethane or ketamine/xylazine anesthesia. Blood–brain barrier (BBB) permeability, cerebral perfusion, and cellular damage were assessed through a cranial window and repeated intravenous injection of fluorescein sodium salt and propidium iodide until 4 h after PT. Neuronal injury and early lesion volume were quantified by stereological cell counting and manual and automated assessment of ex vivo T2-weighted magnetic resonance imaging. Onset SDs originated at the thrombotic core and invaded neighboring cortex, whereas delayed SDs often showed opposite propagation patterns. Seizure induction by 4-aminopyridine caused no increase in lesion volume or neuronal injury in urethane-anesthetized animals. Ketamine/xylazine anesthesia was associated with a lower number of onset SDs, reduced lesion volume, and neuronal injury despite a longer duration of seizures. BBB permeability increase inversely correlated with the number of SDs at 3 and 4 h after PT. Our results provide further evidence that ketamine may counteract the early progression of ischemic injury.

## Introduction

Stroke is the second-leading cause of death and the third leading cause of disability worldwide with a total cost in Europe (2010) of 64.1 billion €.^1,2^ There are currently no proven neuroprotective drugs that improve stroke outcomes. Fundamental challenges to advancing the treatment of stroke are its heterogeneity in terms of cause, pathology, and the lack of mechanistic endpoints in clinical studies. Mechanistic endpoints, necessary for appropriate targeting of treatment, have been lacking due to the limited ability to validate and monitor relevant pathologic processes in clinical populations. Insight into the mechanisms contributing to neural injury and the consequent development of mechanistic endpoints holds great promise to unravel the heterogeneity of stroke, assign appropriate treatment and monitor its efficacy.

Spreading depolarizations (SD) and epileptic seizures have been associated with cerebral ischemia and could serve as mechanistic endpoints.^3–6^ SD is characterized by the near-complete loss of the transmembrane ion-gradients and water influx (=cytotoxic edema).^3,7,8^ Terminal SD, observed in severely ischemic tissue, and transient SDs in less compromised or metabolically intact tissue define a continuum of spreading mass depolarizations.^9,10^ In patients, terminal SDs have been recorded during global cerebral ischemia in the wake of circulatory arrest^11^ and in regions developing new brain infarcts after aneurysmal subarachnoid hemorrhage (aSAH).^5,12,13^ Transient (reversible) SDs have been found in 90–100% of patients with malignant hemispheric stroke (MHS)^14^ and 70–80% of patients with aSAH.^15^ In animals undergoing severe cerebral ischemia, SD characteristically occurs at a spot in the tissue with a delay of 1 min or more following the onset of ischemia.^16^ From there, SD spreads against the gradients of perfusion and oxidative substrate supply into the surrounding increasingly well-perfused tissue. Whereas severely ischemic tissue often remains persistently depolarized, less ischemic and normal tissue recovers from SD. However, further SDs develop in the ischemic penumbra in a recurring pattern that creates the characteristic pattern of temporal clustering.^14,15,17^ Recurrent SDs have been shown to enlarge focal ischemic brain lesions.^18,19^ The common upstream mechanism leading to SD is a reduction of Na^+^/K^+^ ATPase function relative to the demand for pump activity.^3,20,21^

Electrographic seizures were recorded with an incidence of up to 27% in ischemic stroke^22^ and 38% in aSAH patients using continuous non-invasive electroencephalographic (EEG) or invasive electrocorticographic (ECoG) recordings during the first week after the acute insult.^23–26^ While non-convulsive status epilepticus was associated with increased morbidity and mortality following aSAH,^23^ results regarding the impact of seizures on outcome after ischemic strokes have been inconsistent.^4,27^

Here, we compared the impact of SDs and electrographic seizures on the early lesion progression after cortical photothrombosis (PT) in rats. As a secondary outcome parameter, we measured blood–brain barrier (BBB) permeability since SDs and seizures have been shown to increase BBB permeability in animal models^28–30^ and increased BBB permeability predicted neurological outcome after aSAH in patients.^31^ To induce cerebral ischemia, we chose the well-established PT model.^32^ It is characterized by the generation of SDs^32,33^ and by lesion progression.^34^ Frequencies of seizures and SDs were modulated using 4-aminopyridine (4-AP, Kv1 K^+^-channel blocker) and two different types of anesthesia, either urethane or ketamine/xylazine. To measure lesion size, cellular (and specifically neuronal) damage and vascular permeability, we used intravital microscopy,^34,35^ ex-vivo magnetic resonance imaging (MRI), and histopathology.

## Material and methods

All experimental procedures were performed and reported according to the Animal Research: Reporting of In-Vivo Experiments (ARRIVE) guidelines, were approved by the local animal care and ethical committee LAGeSo (Landesamt für Gesundheit und Soziales, G0161/14) and complied with the Charité Animal Welfare Guidelines.

### Photothrombosis model of cerebral ischemia

A total of 30 male Wistar rats (bodyweight 200–430 g (6–11 weeks old; Janvier S.A.S., Germany), housed in groups under a 12-h light-dark cycle with free access to food and water, were deeply anesthetized with intraperitoneal injection of either urethane (1.5–1.8 g/kg bodyweight) or ketamine (80 mg/kg bodyweight) plus xylazine (12 mg/kg bodyweight). As in our previous study,^34^ animals underwent craniotomy over the right somatosensory cortex (2 mm frontal–4 mm occipital and 2 and 6 mm lateral to bregma) and removal of the dura. Thereafter, artificial cerebrospinal fluid (aCSF) containing (in mM) 129 NaCl, 21 NaHCO_3_, 10 glucose, 3 KCl, 1.25 NaH_2_PO_4_, 1.6 CaCl_2_, and 1.8 MgCl_2_ was topically applied to the brain. Oxygen saturation was continuously monitored at the hind paw (Starr Life Science MouseOx probe, Oakmont, PA, USA) and body temperature was kept at 37 ± 0.5°C using a heating pad (Harvard Apparatus, Holliston, MA, USA). PT was induced following cannulation of the tail vein, injection of Rose Bengal (0.133 ml/100 g body weight, 7.5 mg/ml saline, 9.8 µM/kg, Sigma Aldrich, St. Louis, MO, USA) and subsequent focal monochromatic illumination (532 nm, diameter of ∼1 mm, MGL-III-532-5mW-1.5, CNI Laser, Changchun, China) for 15 min. Thereafter, the animal was strictly shielded from light for 1 h to prevent unspecific Rose Bengal activation. The study consisted of three main experimental groups; group 1: PT under urethane anesthesia (*n* = 9), group 2: PT under urethane anesthesia with topical brain surface administration of 4-AP (2 mM, Sigma Aldrich, St. Louis, MO, USA) diluted in aCSF starting 70 min after PT (i.e. after completing fluorescein sodium salt and propidium iodide imaging 1 h after PT) (*n* = 7), and group 3: PT under ketamine/xylazine anesthesia with brain topical administration of 4-AP (500 µM) diluted in aCSF starting 70 min after PT (*n* = 8). Non-ischemic animals (without PT) treated with 4-AP under ketamine/xylazine anesthesia made up a fourth experimental group to examine whether 4-AP would lead to SDs in the absence of PT (*n* = 6).

### Intravital fluorescence and intrinsic optical signal imaging

The imaging-setup consisted of a fluorescence microscope (Zeiss SteReO Lumar V12; Oberkochen, Germany) and a charge-coupled device (CCD) camera (CoolSNAP HQ2, Photometrics, Tucson, AZ 85706, US). Images were captured at ×6.4–×35 magnification and quantitative analysis was performed on ×35 magnification images. Perfusion and BBB permeability assessment were based on intravenous injections of fluorescein sodium salt (1 mg/kg body weight, 1 mg/ml saline, Sigma Aldrich, St. Louis, MO, USA) pre-PT and hourly following PT. Images were taken at 5 Hz for 306 s starting 6 s prior to dye injection. Propidium iodide (Molecular Probes, Eugene, OR, USA; 0.5 mg/kg body weight, 0.5 mg/ml saline) was intravenously injected before and hourly after PT. Quantitative image analysis of propidium iodide was based on an averaged image (consisting of 10 frames, acquisition rate: 1 Hz) acquired 10 min after propidium iodide injection. Intrinsic optical signal (IOS) was visualized by subtracting bright-field images pre-SD from subsequent brightfield images during SD.

### Electrophysiological recordings and laser-Doppler flowmetry

Ion-sensitive microelectrodes inserted to a depth of 200–300 µm from the brain surface were used to simultaneously record direct current electrocorticography (DC-ECoG) and the extracellular potassium concentration ([K^+^]_o_). Electrodes were built in-house from double-barreled theta-glass. The reference side contained 154 mM sodium chloride (NaCl) and the ion-sensitive side contained Potassium Ionophore I 60031 (Fluka, Buchs, Switzerland) at the tip and was filled with 100 mM potassium chloride (KCl) as described previously.^36^ Electrical potentials were converted to K^+^ concentrations using Nernst’s equation and assuming baseline levels of 3 mM [K^+^]_o_. A Power 1401 served for analog-to-digital conversion and Spike 2 software (both Cambridge Electronic Design Limited, Cambridge, UK) was used for recording. Regional cerebral blood flow (rCBF) was continuously recorded using a laser-Doppler probe (Periflux System 5000, Perimed, Järfälla, Sweden) placed on the peri-lesional cortex.

### Ex vivo MRI

At the end of the experiment, animals were perfusion-fixated with 4% paraformaldehyde (PFA). Brains were removed and stored in phosphate-buffered saline (PBS) containing 0.4% PFA. T2-weighted MRI was performed on brains stored in PBS at 7 Tesla (Bruker BioSpec, Ettlingen, Germany) using an 86 mm volume resonator for transmission, a rat brain surface coil for signal reception (Rapid Biomedical GmbH, Rimpar, Germany), Bruker Software (Paravision 6.0.1) and a 2D T2-weighted pulse sequence (2D RARE, repetition time = 8500 ms, echo time = 31.20 ms, rare factor 8, 12 averages, 84 contiguous axial slices per 0.25 mm, field of view 25.6 mm × 25.6 mm, in plane solution 256 × 256; scan time 54 min 24 s). Lesion volume was quantified by two independent approaches of summing up hyperintense voxels in comparison with the contralateral hemisphere: (1) by manually outlining the lesion border in a blinded fashion using Analyze software (AnalyzeDirect, Inc., Stilwell, KS, USA) (performed by KS) and (2) by automated segmentation using an in-house build MATLAB algorithm (MathWorks Inc., Natick, MA, USA) (performed by SL).

### Histology

Following MRI, brains were transferred to cryoprotection solution containing 30% sucrose. Brains were cut in 20 µm thick coronal slices with a cryostat (Leica CM 1950, Wetzlar, Germany) and stained with hematoxylin and eosin following dehydration with increasing ethanol concentration. A Leica DMRA fluorescence microscope (Leica Microsystems, Wetzlar, Germany) with a two-axis computer-controlled stepping motor specimen stage and a CCD camera (Retiga 2000 R, QImaging, Surrey, Canada), as well as the Stereo Investigator 10 software (Microbrightfield Inc., Williston, VT, USA) served for blinded stereological assessment of neuronal damage. Counting was performed under a 40× oil immersion objective by MK and CL. The analyzed region of interest (ROI) included the entire photothrombotic gray matter lesion. In all experiments, the ROI was selected as the ipsilateral gray matter superior to a tangent line through the top of the corpus callosum in each counted slice (Figure 6(b)). The counting of injured neurons was based on their morphological changes following ischemia. These changes include the loss of cytoplasmatic basophilic structures, the shrinkage of the perikaryon into a triangular shape, the presence of edematous changes in the surrounding extracellular space, and the fading or disappearing nucleolus. An injured neuron was defined as a cell lacking a nucleolus with acidophilic cytoplasm, and a shrunken perikaryon surrounded by edematous extracellular space (Figure 6(c)).^37^ We sampled nine slices per brain across the lesion (i.e. one slice every 160 µm), with a counting frame size of 50 × 50 µm and a grid size of 250 × 250 µm (i.e. counting only one 50 × 50 µm frame within each 250 × 250 µm square of the grid). This amount of sampling was sufficient to reach a coefficient of error <0.05, which indicates sufficient reliability of the stereological count estimate.^38^

### Data and image processing

Electrophysiological and imaging data were analyzed using MATLAB. Low-pass filtering (0.5 Hz, DC-signal), band-pass filtering (0.5–45 Hz, alternate current (AC)), and signal power calculation (AC-power) were achieved with build-in functions. Imaging data were corrected for movement artifacts.^35^ Relative perfusion, BBB permeability, and cellular damage were quantified as described previously.^34^ In brief, repeated intravenous fluorescein sodium salt injections and parallel surface imaging were used to estimate perfusion and BBB permeability. First-pass kinetics were used to identify hypoperfused pixels by setting a threshold of 30% of a selected arterial input function (AIF), i.e. the maximal intraarterial first pass image intensity. A pixel-based BBB permeability index was calculated by dividing the integral of the fluorescein signal (excluding the first pass) of each pixel by the time-matched integral of the AIF. Cellular damage was quantified as the number of propidium iodide-positive pixels following background correction by low-pass filtering of the image. BBB permeability and cell damage were assessed as functions of distance to a circular estimate of the lesion border based on the 1 h post-PT angiography.

### Experimental design and statistical analysis

This is an exploratory study. No prior information was available which would have enabled us to perform sample size estimations based on evidence. We thus chose sample sizes which are standard in the field. Animals were excluded in cases of insufficient surgical quality, i.e. if BBB dysfunction or cell damage were detected prior to PT induction. This allowed inclusion of 30 animals to the study. Animals under urethane anesthesia were randomly assigned to 4-AP treatment following PT induction. In vivo imaging data, neuronal cell death, and MRI-based lesion volume were analyzed blindly. For statistical inference, non-parametric Wilcoxon signed-rank test, Mann-Whitney U test or Kruskal–Wallis test (>2 groups) were utilized. Correlations were expressed as Spearman coefficients (rho). Differences were considered significant at *p* ≤ 0.05. Data are shown as median and interquartile range (IQR), i.e. 25th and 75th percentile.

## Results

### PT rapidly elicits SDs but no seizures

**Figure 1.**
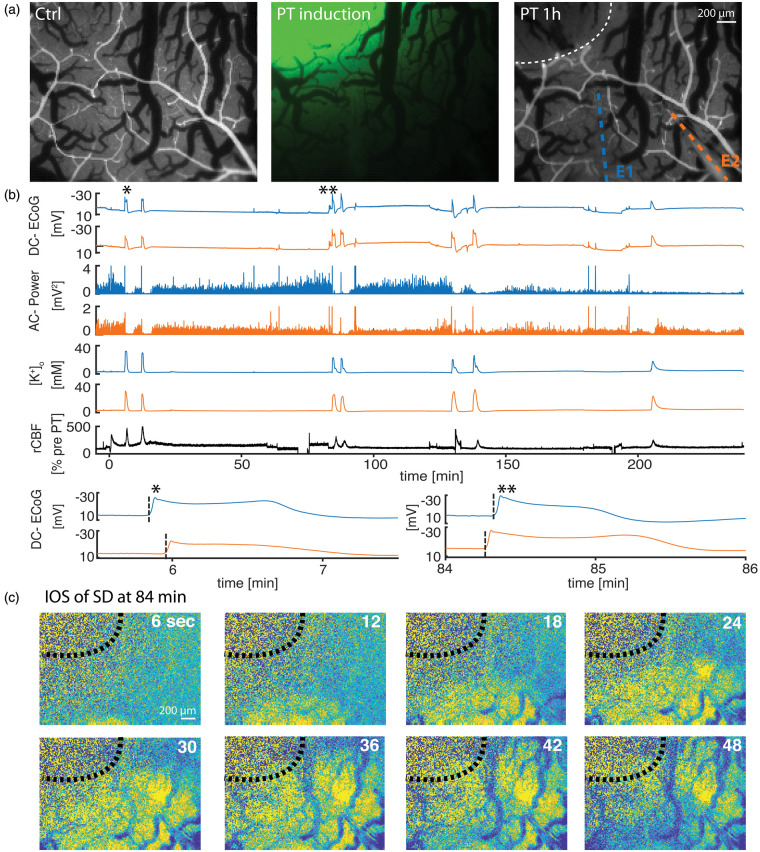
Photothrombosis (PT) model and recordings under urethane anesthesia. (a) Arterial phase of fluorescent angiography prior to PT induction (left), laser spot to activate i.v.-injected Rose Bengal (middle) and fluorescent angiography illustrating occluded vessels in the previously laser-illuminated area (right). Circular dotted line marks lesion border, colored dotted lines overlie the two ion-sensitive glass electrodes. E1: electrode 1; E2: electrode 2. (b) Recordings of one PT experiment under urethane anesthesia from E1 (closer to PT lesion, blue) and from E2 (orange). Each SD (see for example *) displays a transient direct current (DC) potential change (DC-ECoG), a power reduction (alternate current (AC)-power), and a characteristic increase in [K^+^]_o_. SDs induce a hyperemic blood flow response. At higher temporal resolution, the delay of the SD onset between E1 and E2 becomes visible. Note how the initial SD (*) was first recorded at E1, whereas the third SD (**) approximately 84 min after PT was first recorded at E2. (c) Intrinsic optical imaging (IOS) illustrates how the SD wavefront of the third SD originates in peri-thrombotic tissue and propagates towards primarily thrombotic tissue.

**Figure 2. fig2-0271678X20915801:**
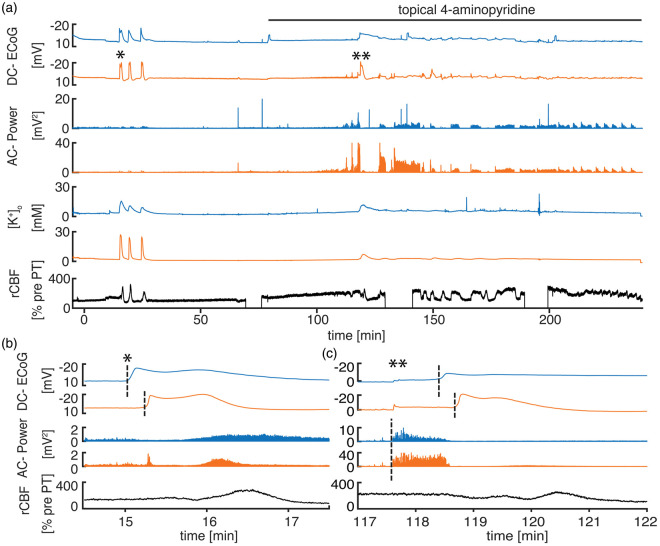
4-AP induces recurrent seizures under urethane anesthesia. (a) Characteristic recording from a PT experiment with pharmacological induction of seizures using 4-AP under urethane anesthesia. 4-AP leads to a gradual increase in AC-power and rCBF, eventually resulting in a first distinct seizure with subsequently alternating ictal activity followed by interictal periods with reduced AC-power. (b) *Onset* SD (*) propagating from the electrode located closer to the thrombotic core to the more remote second electrode with typical negative DC-potential shift, power reduction, and hyperemia. (c) Epileptic seizure without distinguishable delay between electrodes in AC-power recording. With the start of an SD, which here has the same propagation pattern as the *onset* SD, seizure activity is interrupted. The relative hyperemic rCBF response to SD seems reduced because of generally elevated rCBF due to epileptic activity.

**Figure 3.**
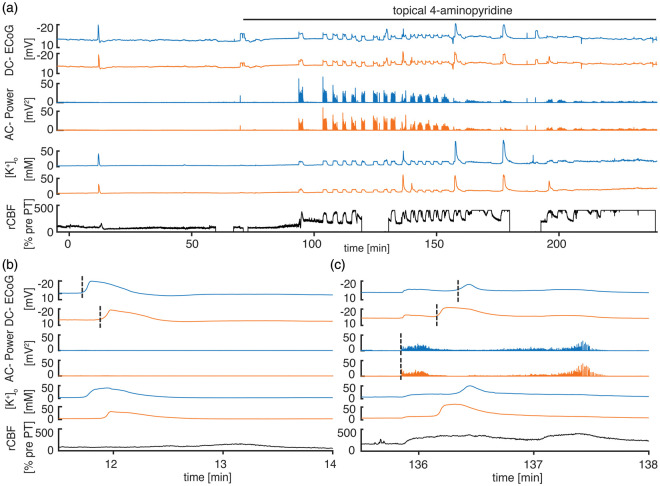
4-AP induces recurrent seizures under ketamine anesthesia. (a) Characteristic recording from a PT experiment with pharmacological induction of seizures using 4-AP under ketamine/xylazine anesthesia. Note that there is only a singular *onset* SD. 4-AP leads to recurrent seizures, and some seizures are associated with SDs. The rCBF response to the *onset* SD and to the epileptic seizures is hyperemic. The rCBF response to delayed seizure-associated SDs is indistinguishable from seizure-induced hyperemia. (b) The *onset* SD propagates from the electrode located closer to the thrombotic core to the second more remote electrode. (c) Seizure onset occurs without distinguishable delay between electrodes in DC-ECoG and AC-power recording. Superimposed SD propagates from the remote electrode to the electrode located closer to the thrombotic core and transiently interrupts epileptic activity. Note the additional negative DC-shift and increase in [K^+^]_o_ when the SD starts during the seizure.

**Figure 4.**
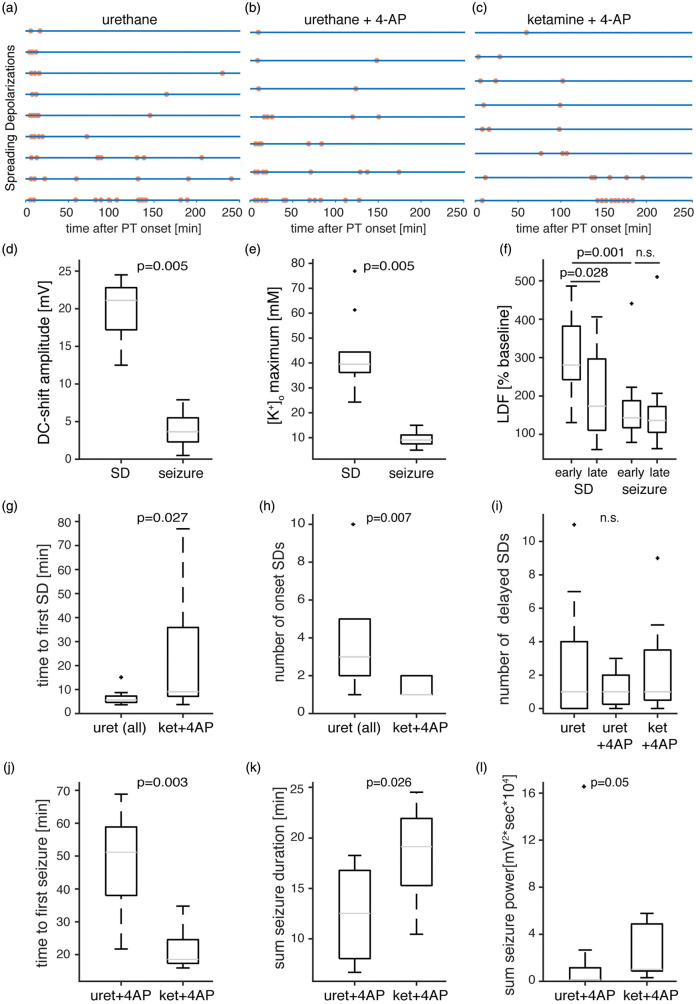
Quantitative seizure and SD analysis. (a–c) Each line represents an animal/experiment and each dot represents a singular SD. Note that *onset* SDs are less frequent under ketamine/xylazine anesthesia. (d–e) Maximal DC-shift amplitudes (d) and maximal [K^+^]_o_ levels (e) are larger during SDs than during seizures (pooled data from groups 2 and 3, *n* = 10, Wilcoxon signed-rank test). (f) The rCBF response to *delayed* SDs is smaller than to *onset* SDs (pooled data from groups 1–3, *n* = 13, Wilcoxon signed-rank test). Early and late seizures evoke similar hyperemic rCBF responses that are smaller than rCBF responses for *onset* SDs (pooled data from groups 2 and 3, *n* = 12, Wilcoxon signed-rank test). (g) The time to the first SD after PT is prolonged under ketamine anesthesia (groups 1 and 2 vs. group 3, *n* = 16 and 8 respectively, Mann–Whitney U test). (h) The number of *onset* SDs is lower under ketamine/xylazine anesthesia (groups 1 and 2 vs. group 3, *n* = 16 and 8 respectively, Mann–Whitney U test). (i) The number of *delayed* SDs is not different across groups (*n* = 9;7;8, Kruskal–Wallis test). (j)The time to the first seizure is shorter under ketamine/xylazine anesthesia (*n* = 7 and 8, Mann–Whitney U test). (k–l)The cumulative seizure duration (K) and seizure power (L) are higher under ketamine/xylazine anesthesia (*n* = 7 and 8, Mann–Whitney U test).

PT, i.e., intravenous injection of Rose Bengal and simultaneous neocortical dye activation with green (532 nm) laser light, resulted in rapid occlusion of blood vessels at the site of illumination (Figure 1(a)). To measure electrographic seizures and SDs, two electrodes were placed at a distance of 340 (IQR: 150–470) µm (*n* = 24) and 870 (550–1100) µm (*n* = 21) from the lesion border (Figure 1(a)). The study consisted of three main experimental groups; group 1: PT under urethane anesthesia (*n* = 9, Figure 1), group 2: PT under urethane anesthesia with 4-AP administered topically 70 min after PT (*n* = 7, Figure 2), and group 3: PT + 4-AP under ketamine/xylazine anesthesia (*n* = 8, Figure 3). SD was recorded in all animals and the first SD occurred at a median of 5.6 (4.6–7.3) min following Rose Bengal injection under urethane anesthesia (groups 1 and 2, *n* = 16; Figure 1(b)) and was termed *onset SD* because of the close temporal proximity to PT induction. In 13 of 16 animals (groups 1 and 2), the *onset* SD was followed (within minutes) by up to four additional SDs to form an *onset complex* of SDs (Figure 4(a) and (b)). SDs were identified by their typical features: a negative DC potential shift, a concomitant rise in [K^+^]_o_, and depression of the AC-ECoG activity in electrically active tissue (=spreading depression).^39^ *Onset* SDs originated at the thrombotic region and invaded the surrounding cortex. SDs were termed *delayed* SDs from the start of 4-AP perfusion in groups 2 and 3. Accordingly, all SDs starting later than 70 min after PT induction were termed *delayed* SDs in group 1, wherein no 4-AP was administered. In animals that displayed *delayed* SDs, they spread in reverse direction (i.e. from the periphery to the thrombotic center) in 12 out of 16 experiments (pooled experimental groups) as revealed by the electrophysiological recordings and IOS imaging (Figure 1(c)). Spontaneous seizures were not recorded in any of the animals during the 4 h monitoring after PT under urethane anesthesia in group 1 or in any of the animals during the first 70 min before 4-AP perfusion under either urethane or ketamine/xylazine anesthesia (groups 2 and 3).

### 4-AP-induced electrographic seizures show smaller DC-amplitudes and peak [K^+^]_o_-levels than SDs

To test the effect of seizures on injury progression, we applied the convulsant Kv1 K^+^-channel blocker 4-AP starting 70 min after PT in groups 2 and 3. 4-AP application resulted in a gradual increase in AC-ECoG power that eventually turned into distinct recurrent seizures (Figure 2(a)). The maximal negative DC-shift as well as the peak [K^+^]_o_ levels during electrographic seizures were significantly smaller than during SDs (seizures: −3.7 (−5.5−(−2.3)) mV and 9.1 (7.5–11.1) mM vs. SDs: –21.1 (–22.8–(–17.2)) mV and 39.5 (36.2–44.4) mM; negative DC-shift and peak [K^+^]_o_ respectively, *n* = 10, *p* = 0.005, Wilcoxon signed-rank test, pooled data from groups 2 and 3, Figure 4(d) and (e)). [K^+^]_o_ between recurrent SDs and/or seizures increased from 3 to 4.5 (4.0–6.5) mM during the fourth hour of recording (all groups, *n* = 19, *p* < 0.001, Wilcoxon signed-rank test) without significant differences between seizing (groups 2 and 3) and seizure-free (group 1) animals (*p* = 0.45, *n* = 10;9; Mann–Whitney U test). Return of [K^+^]_o_ to pre-SD levels was slower or less complete after delayed SDs than after *onset* SDs (Figures 1 [Fig fig2-0271678X20915801]to 3).

### Suppression of SD and lower seizure threshold under ketamine

To investigate the effect of SD on injury progression, animals were anesthetized with ketamine and xylazine in group 3 (Figure 3), since ketamine was previously shown to block propagation and, at higher doses, initiation of SDs.^40^ Under ketamine, SDs were still recorded in all animals (Figure 4(c)), yet the latency to the first SD following PT was significantly longer compared with animals under urethane anesthesia (group 3: 9.1 (7.2–35.9) min, *n* = 8, vs. pooled groups 1 and 2: 5.6 (4.6–7.3) min, *n* = 16, *p* = 0.027, Mann–Whitney U test, Figure 4(g)). Further, the number of *onset* SDs was lower under ketamine (group 3: 1 (1–2) SD, *n* = 8, vs. pooled groups 1 and 2: 3 (2–5) SDs, *n* = 16, *p* = 0.007, Mann–Whitney U test, Figure 4(h)). The number of delayed SDs was similar in all groups (group 1: 1 (0–4), *n* = 9, group 2: 1 (0.25–2), *n* = 7, group 3: 1 (0.5–3.5) SD, *n* = 8, *p* = 0.97, Kruskal–Wallis test, Figure 4(i)).

The seizure threshold was lower under ketamine/xylazine anesthesia than under urethane. While topical application of 4-AP at a concentration of 500 µM reliably induced recurrent seizures under ketamine (see also Prager et al.^30^), under urethane anesthesia, recurrent seizures were inducible only at 2 mM 4-AP and the latency to a first seizure after application of 4-AP was longer (urethane (group 2): 51.1 (38.0–58.9) min, *n* = 7, compared with ketamine (group 3): 18.5 (17.4–24.6) min, *n* = 8, *p* = 0.003; Mann–Whitney U test, Figure 4(j)). Cumulative seizure duration and power were higher under ketamine than urethane (ketamine (group 3): 19.1 (15.3–21.9) min and 1.02 (0.88–4.88) 10^4^ × mV^2^s, *n* = 8, vs. urethane (group 2): 12.5 (8.0–16.8) min and 0.13 (0.12–1.16) 10^4^ × mV^2^s, *n* = 7, *p* = 0.026 and *p* = 0.05, Mann–Whitney U tests, Figure 4(k) and (l)). Following 4-AP, SDs were often coupled with seizures and interrupted seizure activity (Figure 3(a) and (c)). No SDs were recorded when 4-AP was applied under ketamine anesthesia without prior PT induction (non-ischemic animals, *n* = 6).

### Reduced rCBF responses to delayed SDs, but not to seizures

The blood flow response to SD (measured by LDF located near the lesion) was characterized by hyperemia. Regional CBF increased by 280 (242–382)% compared to pre-PT baseline during *onset* SDs. Hyperemia was reduced to 173 (111–297)% (*p* = 0.028, *n* = 13, Wilcoxon signed-rank test, pooled data from groups 1 to 3, Figure 4(f)) in response to SDs recorded during the last (i.e. 4th) hour of recording. In contrast, rCBF increases during seizures were smaller and similar between the first and the last recorded seizures (LDF early seizure: 143 (117–188)%; late seizure: 136 (105–173)% (*n* = 12, *p* = 0.43, Wilcoxon signed rank test, pooled data from groups 2 and 3, Figure 4(f)).

### Intravital microscopy reveals progressive hypoperfusion, increased BBB permeability and cellular damage

**Figure 5.**
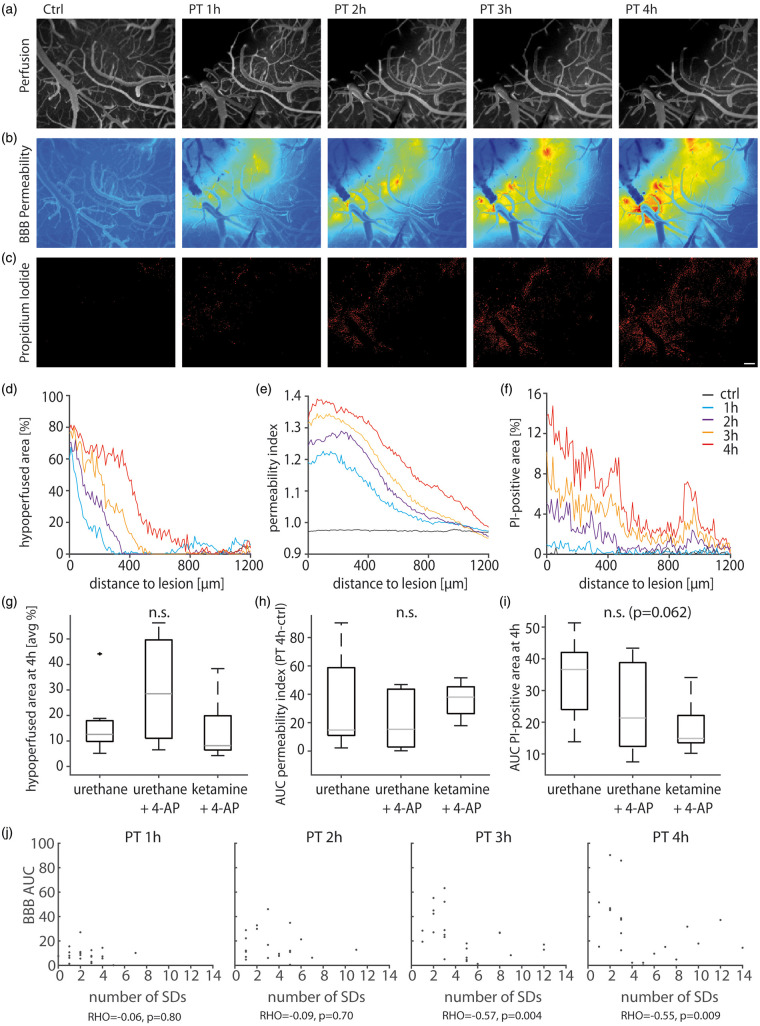
In-vivo imaging of cerebral perfusion, BBB permeability, and propidium iodide. (a) Arterio-venous overlay of fluorescent angiography before and 1–4 h after PT. Note that the hypoperfused (dark) area grows with time. (b) Fluorescein extravasation relative to the intravascular tracer signal reveals a growing area with BBB dysfunction between 1 and 4 h after PT. (c) Propidium iodide (PI) staining reveals a growing number of cells with membrane damage. Scalebar = 200 µm. (d) Graphical illustration of the hypoperfused area relative to the primary lesion shows a growing perfusion deficit. (e) Similarly, the amount of tissue affected by BBB dysfunction and (f) the propidium iodide-positive area grows with time. (g–i) Quantitative analysis shows no difference in perfusion (*n* = 9;7;7, Kruskal–Wallis test) and BBB permeability (*n* = 9;7;7, Kruskal–Wallis test) across groups and a trend towards reduced propidium iodide-signal (*n* = 9;7;7, Kruskal–Wallis test) under ketamine/xylazine anesthesia. (j) BBB permeability inversely correlates with the number of SDs at 3 and 4 h after PT.

To quantify lesion progression, we measured perfusion, BBB permeability and cell damage using intravital microscopy. Fluorescein-based angiography demonstrated complete arterial and venous occlusion in the laser-illuminated cortical region (Figure 5(a)). As time progressed, the initial perfusion deficit (as defined 1 h after PT) expanded into primarily well-perfused cortex (Figure 5(a) and (d)). Extravasation of i.v.-injected fluorescein indicated increased BBB permeability (Figure 5(b) and (c)). Concomitantly, the propidium iodide signal increased in the cortical area surrounding the primary perfusion deficit (Figure 5(e) and (f)). Neither perfusion nor BBB permeability were different between groups (Figure 5(g) to (i)), yet a trend towards a reduced propidium iodide signal was observed under ketamine (*p* = 0.06, Kruskal–Wallis; Figure 5(i)). The propidium iodide signal after 4 h as measured directly at the recording site (diameter of 100 µm around the electrode tip) did not correlate with the number of SDs, but with the counted injured neurons (see next paragraph) for electrodes located <400 µm from the lesion border (Spearman’s rho = 0.58, *p* = 0.040, *n* = 14). No correlation was found for more remote electrodes (Spearman’s rho = 0.24, *p* = 0.363, *n* = 17). The 400 µm distinction is based on perfusion data, i.e. this is the zone which was shown to undergo early progressive hypoperfusion in our previous study.^34^ Interestingly, BBB permeability negatively correlated with the number of SDs at 3 and 4 h after PT when data from all three groups were pooled (Spearman’s rho=−0.57 and −0.55, *p* = 0.004 and 0.009, *n* = 23, Figure 5(j)). No correlations were found between BBB permeability and the number of seizures, cumulative seizure power or accumulation of [K^+^]_o_ in the 4-h-monitoring period after PT.

### Ketamine anesthesia was associated with reduced lesion size and neuronal damage

**Figure 6.**
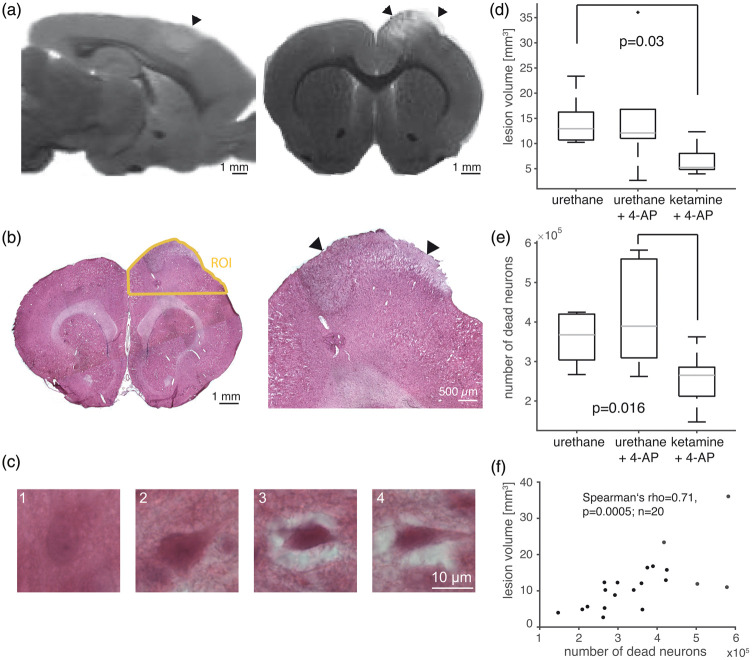
Ex-vivo assessment of lesion volume and neuronal damage. (a) Sagittal and coronal section of ex vivo T2-weighted MRI with arrowheads pointing to the thrombotic lesion. (b) Hematoxylin and eosin staining of coronal section. Yellow line shows the region of interest (ROI) used for stereological cell counts. Arrowheads point to the thrombotic lesion. (c) Illustration of morphological criteria used to count injured neurons. A neuron with oval perikaryon, a nucleolus and homogenous extracellular matrix (1). Dark perikaryon and loosened extracellular space (2). Dark and shrunk perikaryon in edematous extracellular space (3). Triangular, dark and shrunk perikaryon in edematous extracellular space (4) Neurons fulfilling criteria 3 and 4 were counted as injured. (d) MRI-based lesion volume was reduced under ketamine/xylazine anesthesia compared with the seizure-free urethane group (*n* = 7;6;7, Kruskal–Wallis test). (e) Fewer injured neurons were counted under ketamine/xylazine anesthesia compared with animals under urethane anesthesia and induction of seizures (*n* = 8;7;7, Kruskal–Wallis test). (f) MRI-based lesion volume assessment correlated with stereological counts of injured neurons.

To assess cortical lesion volume and neuronal damage, brains were removed ∼4.5 h after PT. On manually segmented ex-vivo T2-weighted MRI (Figure 6(a)), animals with 4-AP-induced seizures under urethane anesthesia did not show an increased lesion size (number of hyperintense voxels ipsi- vs. contralateral) compared to seizure-free animals (group 2: 12.1 (11.0–16.8) mm^3^, *n* = 6, vs. group 1: 12.9 (10.7–16.2) mm^3^, *n* = 7). In contrast, lesion size was reduced to 5.3 (4.8–8.0) mm^3^ in animals under ketamine anesthesia (group 3, *n* = 7, *p* = 0.03; Kruskal–Wallis test, Figure 6(b)) despite the occurrence of seizures. To validate manual lesion volume measurements, we employed an automated approach for detection of hyperintense voxels and found a positive correlation between these two independent approaches (Spearman’s rho 0.61, *p* = 0.005, *n* = 20). Blind stereological counting of apoptotic/necrotic neurons (see Methods and Figure 6) revealed similar neural injury in the cortex of animals under urethane anesthesia with or without seizures (group 1: 3.68 (3.04–4.20) ×10^5^ and group 2: 3.89 (3.09–5.60) ×10^5^ counted neurons), whereas the number of injured neurons was lower under ketamine anesthesia (group 3: 2.65 (2.12–2.86) ×10^5^ neurons, *p* = 0.016, Kruskal–Wallis test, *n* = 8;7;7 animals, Figure 6(d)). MRI-based lesion volume assessment correlated with stereological cell counts (Spearman’s rho = 0.71, *p* = 0.0005, *n* = 20, Figure 6(e)).

## Discussion

In the present study, we compared the impact of the two network pathologies – SDs and electrographic seizures – on early ischemic lesion progression. Combining electrophysiological measurements with in-vivo imaging of cerebral perfusion, BBB permeability and cellular injury as well as ex-vivo assessment of lesion size (MRI), and neuronal injury (histopathology), we found that: (1) SDs abundantly occur following PT; (2) ketamine anesthesia was associated with reductions of (a) *onset* SDs, (b) early lesion volume and (c) early neuronal injury; (3) seizures did not occur spontaneously in presence of the antiepileptic drugs urethane and ketamine; (4) when pharmacologically triggered by 4-AP, seizures did not alter BBB permeability, propidium iodide signal, MRI-proven lesion volume or histopathological neuronal injury; and (5) BBB permeability negatively correlated with the number of SDs at 3 and 4 h after PT induction.

SDs are common in the PT model.^33^ In a longitudinal in-vivo MRI study of this model in rats, they were associated with reductions of the apparent diffusion coefficient (ADC), indicating cytotoxic edema.^41^ The ADC reduction became rapidly irreversible in the illuminated thrombotic cortex and was progressively irreversible in a surrounding rim of tissue, which is regarded as the ischemic penumbra in the PT model^41^ (cf. literature^18,42–44^ for the discussion of penumbra definitions). Hence, in addition to many other models and recent clinical data, also the PT model has provided evidence for van Harreveld’s original hypothesis,^45,46^ as reviewed recently,^7^ that SD is the electrophysiological correlate of the cytotoxic edema in the brain’s gray matter. The present study confirms earlier results that characterized the ischemic penumbra as a growing region of local hypoperfusion with progressive dysfunction of the BBB.^34^ The observation that delayed SDs typically originated from initially non-ischemic, peri-thrombotic tissue further supports on-going lesion progression. A previous MCAO study in mice found that such *delayed* SDs depended on transient worsening of mismatch between oxidative substrate supply and demand. Such transient worsening could result not only from systemic arterial blood pressure fluctuations but also, for example, from local neuronal activation in the unstable tissue.^47^ Another observation indicating progressive metabolic compromise was the prolonged duration of *delayed* SDs compared with *onset* SDs accompanied by a slower normalization of [K^+^]_o_ after the *delayed* SDs and increasingly elevated baseline levels of [K^+^]_o_ between recurrent SDs or seizures.^9,39^ The elevated baseline level of [K^+^]_o_, in turn, reduces the activity of the α_2_/α_3_-portion of the sodium-potassium ATPase.^48^ This might additionally contribute to the prolongation of SDs and SD-related cytotoxic edema, thereby facilitating lesion progression.

Consistent with the inextricable connection between SD and cytotoxic edema, we found a lower number of *onset* SDs associated with a smaller lesion volume and less extensive neuronal injury at sacrifice after 4.5 ho in ketamine/xylazine versus urethane-anesthetized rats. Ketamine is a non-competitive antagonist of the ionotropic N-methyl-D-aspartate receptor (NMDAR) with an SD-inhibiting effect both in lissencephalic^49–52^ and gyrencephalic animals.^40,53^ In addition, high-dose S(+)-ketamine, the two times stronger, active enantiomer of ketamine, improved neurological outcome following incomplete cerebral ischemia due to combined right common carotid artery occlusion and hemorrhagic hypotension in rats.^54^ High-dose S(+)-ketamine also reduced cortical neuronal cell loss at sacrifice six days after global forebrain ischemia in rats.^55^ In our experiments, xylazine, an alpha 2 adrenergic agonist, may have exerted additional neuroprotective effects.^56^

MK-801, another non-competitive NMDAR antagonist, has been studied more extensively than ketamine. It acts at the same binding site but is more potent.^51^ Accordingly, MK-801 also inhibits SDs in rats, as shown for KCl-triggered SDs propagating through naïve tissue, but also for SDs induced by either MCAO or brain topical application of the vasoconstrictor endothelin-1.^57–60^ In parallel, MK-801 protects from ischemic damage as summarized in a recent meta-analysis.^61^ There are, however, a number of caveats. According to a previously described dose–response curve for neuroprotection by MK-801,^62^ lower doses (0.5 and 1 mg/kg body weight i.p.) protected the brain from ischemic damage in some,^63–66^ but not all studies,^67–69^ whereas a medium-dose of MK-801 (3 or 5 mg/kg body weight intraperitoneally (i.p.)) robustly reduced ischemic lesion volume when measured at sacrifice either 3 or 24 h after MCAO in rats^58,59,70^ and 6 h after MCAO in cats.^71^ Importantly, low-dose treatment with MK-801 blocked the spread of SDs into the adequately supplied surrounding tissue, but failed to inhibit SDs in the ischemic penumbra and did not protect from ischemic damage.^68^ Not only a sufficient dosage, but also the anesthetic, against which NMDAR antagonists are tested, can complicate the interpretation of the results. The neuroprotective effect might be rather small or absent when ketamine or MK-801 are tested against isoflurane, which competitively inhibits NMDARs at the glycine site,^72^ suppresses SDs,^73,74^ increases rCBF, and presumably for these reasons are also neuroprotective.^70,75,76^ A clear limitation of the treatment with NMDAR antagonists is that they cannot block terminal SD in severely ischemic tissue even at the highest concentrations and are thus unable to protect this tissue.^77–80^ One potential explanation is the additional involvement of non-NMDA glutamate and GABA_A_ receptors in SD during severe ischemia.^9^ In the ischemic penumbra, NMDAR antagonists can only partially inhibit SDs at high doses. Accordingly, *onset* SDs occurred less frequently after PT when ketamine was administered than when urethane was administered. Nevertheless, *onset* SDs still occurred under ketamine. Regarding the relative resistance of the penumbra to NMDAR antagonists, it is interesting to note that the progressive increase of the baseline [K^+^]_o_ level alone into a range typically observed in the ischemic penumbra was sufficient to render SDs increasingly resistant to MK-801.^60^

The main practical problem of NMDAR antagonists such as MK-801 or ketamine is that they lead to psychosis at lower and to coma at higher doses^81^ at which they partially inhibit SDs in the penumbra and therefore are neuroprotective. Avoiding psychosis and maintaining wakefulness are, without doubt, important goals in alert patients with stroke,^39^ restricting treatment of this patient population to low doses of NMDAR antagonists, at which they do not inhibit SDs in the penumbra.^68,82^ Accordingly, the ensuing clinical trials which exclusively investigated the effect of low doses showed neither significant benefit nor harm from NMDAR antagonists.^83,84^

The renewed interest in NMDAR antagonists is now derived from two parallel clinical developments. First, ketamine has found its way into routine neurocritical care practice as a second-line combination treatment together with the GABA_A_ agonist midazolam to sedate patients with severe acute cerebral injuries. In this patient population, ketamine is often administered at higher doses because of its beneficial cardiocirculatory and bronchodilatory properties.^85,86^ Second, ketamine has been associated with a decrease in SD incidence in a mixed population of 60 patients with traumatic brain injury (TBI), 31 patients with aSAH and 24 patients with malignant hemispheric stroke.^87^ The first prospective controlled trial of ketamine for SD inhibition recently confirmed this observation in eight patients with TBI and two with aSAH.^88,89^ In addition, a case-report of spontaneous intracerebral hemorrhage demonstrated suppression and re-appearance of a cluster of SDs in response to intermittent ketamine treatment.^90^ In a recent case series of 66 aSAH patients, low- and high-dose administration of s-ketamine was retrospectively compared. The high-dose range was above the upper limit for sedation recommended by the manufacturer but resulted in further significant decrease in SD incidence.^91^ The present experimental study provides further support for the use of ketamine and midazolam – rather than propofol and midazolam^92^ – as first-line combination treatment in neurocritical care when clusters of SDs are recorded, as it suggests that ketamine inhibits SDs in the penumbra and is neuroprotective on this account. Notwithstanding its challenges, this concept should be further tested in a neuromonitoring-guided, randomized, multicenter clinical trial.

In addition to SDs, electrographic seizures could be a therapeutic target to reduce neuronal damage after ischemia. In the rat MCAO model, electrographic seizure rates within the first hour ranged between 28 and 81% under isoflurane and halothane anesthesia, respectively.^5,93^ In the neocortical PT model, hippocampal seizures were recently recorded in 66% of the rats during the first week.^94^ However, we did not detect electrographic seizures during the 4-h monitoring period, unless the proconvulsant 4-AP was given. This could be due to intrinsic differences between the MCAO and the PT model. Especially transient MCAO models create a large volume of penumbral tissue, whereas the PT model is characterized by a more rapidly evolving ischemic core surrounded by only a small penumbra. This may be related to differences in collateral perfusion.^34,95^ Consequently, in the acute phase after MCAO, there may be more vital yet dysfunctional tissue showing aberrant (epileptic) network activity. In addition, antiepileptic properties of both urethane and ketamine, which were shown to inhibit epileptic activity in animal models,^96–103^ have likely prevented seizures in our experiments. Specifically in the 4-AP model (given i.p.), ketamine prevented generalized tonic-clonic seizures in ∼40% of the animals and delayed their onset in the remaining animals.^103^ In patients, ketamine has been successfully used as a third- or fourth-line treatment of refractory status epilepticus.^101,104–106^ In the present study, animals under urethane anesthesia showed a higher threshold for seizure induction by 4-AP than animals under ketamine, indicating stronger antiepileptic properties of urethane than ketamine at typical anesthetic doses.

In the present animal study, artificial induction of seizures during the early time window had no effect on early ischemic lesion progression. The early sacrifice at 4.5 h after onset of PT is a limitation of our study as the final lesion size was not evaluated and harmful effects of seizures may not become apparent until later. Another limitation is that we could not investigate the effect of seizures or SDs on lesion progression in the absence of an anesthetic.

As a secondary outcome variable, we investigated BBB permeability. Increased BBB permeability is associated with poor outcome in several brain disorders^107,108^ including, for example, aSAH.^31^ Consistent with previous studies that found BBB dysfunction as early as 5 min after PT in the thrombotic core,^34,109,110^ we observed early BBB dysfunction at 1 h (the earliest investigated timepoint), i.e. several hours earlier than after MCAO.^111–114^ This is likely explained by the different mechanisms of stroke induction in these models. PT involves the generation of free radicals during Rose Bengal activation which leads to rapid damage to the endothelium in the thrombotic core.^109^ However, whether free radicals are involved in the further progression of BBB dysfunction in the peri-thrombotic tissue is less clear. Thus, topical application of a free radical scavenger in combination with an inhibitor of nitric-oxide synthase 30 min after PT did not reduce the expansion of BBB dysfunction into peri-thrombotic tissue.^34^ This may suggest different mechanisms of BBB dysfunction in the thrombotic core and peri-thrombotic tissue. However, upregulated transcytosis seems to be the common pathway of BBB dysfunction in both thrombotic core and peri-thrombotic tissue. This was evidenced by protein-tracer detected in endothelial cells 5 min after PT in the core^109^ and by an increased number of endothelial caveolae and vacuoles 3 h after PT in peri-thrombotic tissue while tight junctions were mainly intact.^115^ Opening of the transcellular before the paracellular pathway also occurs after MCAO^114,116^ and in response to many other challenges such as, for example, intracarotid injection of mannitol or brain topical dehydrocholate.^117^

In a study in non-ischemic cortex of mice and rats exposed by bilateral craniotomy, pinprick-induced SDs led to ipsilateral Evans blue extravasation after a delay of 3 h. This was attributed to paracellular leakage mediated by degradation of tight junction proteins by matrix-metalloproteinase 9.^29^ In another study in mice, SDs induced caveolin-dependent transcellular extravasation of Evans blue remote from the site of KCl application with a delay of 6 h.^28^ Hence, BBB dysfunction in peri-thrombotic tissue occurs 2–5 h earlier than following SDs in the described non-ischemic animals. This suggests that early BBB dysfunction is triggered by mechanisms other than SD. In line with this conclusion, BBB dysfunction after PT, as assessed by gadofluorine M-enhanced MRI was unaltered by treatment with MK-801.^110^

Incidentally, we found a negative correlation between the number of SDs and the increase in BBB permeability 3 and 4 h after PT. The increase in BBB permeability in the penumbra causes accumulation of proteins such as albumin in the extravascular extracellular space. Following Gibbs–Donnan forces, this should lead to the uptake of water into the extracellular space. The larger extracellular space should dilute ions, metabolites, and neuroactive substances released from the cells^118^ and this could possibly protect against further SDs.^7^

## Conclusion

ECoG neuromonitoring in patients provides the option for early treatment stratification according to SDs detected in real time, and then to record the response to a neuroprotective intervention. A pressing clinical question is whether sedation-requiring patients displaying clusters of SDs in the wake of acute cerebral injuries should receive a combination of sedatives including ketamine in order to inhibit SDs in the penumbra. The present results in the photothrombosis stroke model provide further momentum to test this concept in a neuromonitoring-guided, randomized, blinded, multicenter, feasibility trial.
